# Towards environmental management of WEEE in Brazil: evaluating the impacts of recycling plastics

**DOI:** 10.1007/s11356-026-37732-w

**Published:** 2026-04-16

**Authors:** Abner Fernandes Souza da Silva, Guilherme Andreazza de Freitas, Diogo Aparecido Lopes Silva, Jane Maria Faulstich de Paiva, Juliana Mendes Campolina, Virgínia Aparecida da Silva Moris

**Affiliations:** Federal University of Sao Carlos - Campus Sorocaba: Universidade Federal de Sao Carlos - Campus Sorocaba, Sorocaba, Brazil

**Keywords:** Life cycle assessment, Plastic recycling, Electronic waste, WEEE, Environmental impact, ABS, HIPS

## Abstract

Plastic fractions recovered from waste electrical and electronic equipment (WEEE) are growing rapidly worldwide, yet their net environmental benefit remains under-quantified—especially in emerging economies such as Brazil, where recycling infrastructure is still maturing. Acrylonitrile–Butadiene–Styrene (ABS) and High-Impact Polystyrene (HIPS) together account for a large share of this stream and offer high material value if efficiently reprocessed. This study applies ISO 14040/14044 life cycle assessment to quantify the impacts of recycling ABS and HIPS collected from Brazilian WEEE. Foreground data from an industrial plant in Sorocaba were modeled with ReCiPe 2016 midpoint indicators. Extrusion emerged as the dominant hotspot, contributing 75.2% (ABS) and 85.7% (HIPS) of total impacts, whereas transport accounted for < 5%. Cutting extrusion energy by 30% reduced global warming potential (GWP) by 25% for HIPS and 22% for ABS, while switching to Brazil’s renewable-rich grid reduced impacts across the main categories. Compared with virgin resin, recycling resulted in substantially lower global warming impacts for both HIPS and ABS. The sensitivity analysis showed that adopting an economic allocation approach led to lower environmental impacts compared with mass-based allocation for both ABS and HIPS. Optimizing extrusion energy and adopting low-carbon electricity are therefore critical to maximizing the environmental benefits of WEEE-plastic recycling. Beyond technical insights, these results provide evidence to guide public policies that promote recycling infrastructure and energy efficiency, while reinforcing that recycled plastics offer substantially lower environmental burdens compared with virgin resources.

**Supplementary Information:**

The online version contains supplementary material available at 10.1007/s11356-026-37732-w.

##  Introduction

Waste Electrical and Electronic Equipment (WEEE) is the fastest-growing waste stream globally, with an annual growth rate of around 3 to 5% (Liu et al. 2023). This growth is driven by increasing consumption of Electrical and Electronic Equipment (EEE), coupled with shorter product lifespans and a growing trend towards replacement rather than repair and reuse (El-Sherif et al. 2024). According to Baldé et al. (2024), 62 million tons of WEEE were generated worldwide in 2022, compared to 53.6 million tons in 2019, reflecting a 15.6% increase over four years. Given this rapid growth, the disposal of WEEE has become a significant concern in many countries (Mulya et al. 2022), particularly due to its complex composition, which includes plastics, ferrous and precious metals, heavy metals, flame retardants, and fiberglass. When not properly managed, these materials can lead to severe environmental impacts, such as soil and water contamination, as well as significant risks to human health (Twagirayezu et al. 2022). Furthermore, improper disposal results in the loss of valuable resources that could be reintegrated into the production chain, providing economic benefits (Pokhrel et al. 2020; Pryshlakivsky and Searcy 2021). Therefore, WEEE recycling is an important activity to recover essential raw materials (Palanisamy and Subburaj 2023).

Among the various materials present in WEEE, plastics deserve particular attention due to their long decomposition time, significant landfill space occupation, and high potential for reuse in other products before final disposal (Mendes Campolina et al. 2017). Plastics make up approximately 20 to 30% of WEEE, making their recovery particularly attractive (Ardolino et al. 2021; Andrianisa et al. 2024). In response to the growing challenges associated with WEEE, several regulatory frameworks have been established worldwide. In Europe, two primary legislations address this issue: 2002/95/EC and 2002/96/EC, which restrict the use of certain hazardous substances in EEE and promote WEEE management through reuse, recycling, and other treatments, respectively (European Union 2003a, 2003b). In Brazil, the focus of this study, the National Solid Waste Policy was established in 2010, mandating shared responsibility among all supply chain entities for the proper disposal of solid waste through reverse logistics, including WEEE (Brasil 2010). However, it was only in 2020 that a new law emerged, setting goals and responsibilities for companies in the sector regarding transparency in management (Brasil 2020). According to the law, Brazil must collect and recycle 17% of all EEE sold in the country by 2025.

In addition, the implementation of the National Circular Economy Strategy (ENEC) (2024) represents a milestone in the transition from the linear model to a circular economy (CE). This strategy emphasizes the recovery and reintegration of materials into production cycles, guided by principles such as waste elimination, material circulation, and environmental regeneration. Accordingly, ENEC also establishes guidelines that reinforce these principles while also encouraging the creation of markets for recycled products and the establishment of recycling facilities throughout the country. By promoting an integrated approach, ENEC aims to align public policies, international commitments, and private initiatives, thereby creating a regulatory and financial environment favorable to circular practices such as recycling (Brasil 2024a). In parallel, the Recycling Incentive Law (LIR) (Brasil 2024b) establishes tax incentives and benefits for projects that stimulate the recycling value chain, including the creation of Investment Funds for Recycling Projects (ProRecicle). An ordinance issued in 2024 further defined the procedures for submission, evaluation, monitoring, and accountability of such initiatives. Despite the various benefits, reverse logistics presents several barriers to implementation, such as variable quality of collected products, low predictability of collection routes, low volume, high costs, planning difficulties, limited visibility, and financial aspects (Rogers and Tibben-Lembke 1998; Teixeira et al. 2023).

In this context, Life Cycle Assessment (LCA) is a tool that can help propose more environmentally appropriate disposal alternatives for WEEE (Cesaro et al. 2024; Nikolic et al. 2024). LCA evaluates the environmental performance of a product, service, or process throughout its entire life cycle, from raw material extraction to final disposal (Finnveden et al. 2009). Several studies have addressed the use of LCA in WEEE management, highlighting the environmental benefits of proper recycling and resource recovery across different contexts. For example, Rocha and Penteado (2021) studied the reverse logistics of WEEE in the Campinas region of Brazil, concluding that environmentally correct disposal of WEEE provides more benefits than environmental impacts and can offer various gains. Similarly, Islam and Iyer-Raniga (2023) studied the management of waste Printed Circuit Boards (PCB) in Australia, comparing recycling in other countries with hypothetical scenarios considering all treatment being done locally. Their results showed that recovering metals and incinerating the remainder for energy recovery resulted in the lowest overall environmental burden. Ismail and Hanafiah (2021) conducted a similar study in Malaysia and concluded that incineration was the least environmentally impactful option for the studied context. In another context, Pokhrel et al. (2020) studied the environmental and economic feasibility of recovering precious metals from printed circuit boards and concluded that gold is the most significant element in both aspects.

Other studies have focused on operational aspects and management scenarios. Ghiga et al. (2023), for instance, analyzed three WEEE management scenarios in Iasi, Romania, using the LCA methodology, concluding that collection has the greatest environmental impact, emphasizing the importance of awareness campaigns to improve collection and recycling efficiency. The three scenarios represent progressive approaches to WEEE management, ranging from basic collection systems with limited public participation to advanced systems involving multiple stakeholders, awareness campaigns, and incentive mechanisms, achieving significantly higher collection rates. Borrirukwisitsak et al. (2023) used Material Flow Analysis (MFA) and LCA methodologies to assess the environmental impacts of manual WEEE dismantling in Thailand. They concluded that recycling valuable parts can significantly reduce negative environmental impacts, highlight the importance of proper management and increase recyclable parts in the waste stream.

Other studies have analyzed WEEE plastic recycling using various methods, including LCA. Spirio et al. (2024) highlighted the application of a valorization strategy for WEEE plastics, specifically Polypropylene (PP), for producing filaments suitable for 3D printing processes by Fused Filament Fabrication (FFF). The methodology involved analyzing and separating plastic waste, optimizing extrusion conditions, and evaluating the rheological and mechanical properties of the resulting filaments. The main result indicated that PP-WEEE filaments, when properly processed, exhibit properties comparable to commercial filaments, demonstrating the feasibility of using recycled WEEE plastics in high-quality additive manufacturing. Gaikwad et al. (2018) applied LCA in a similar process and concluded that using WEEE plastics for this purpose can result in a 30% reduction in Greenhouse Gas (GHG) emissions. Teixeira et al. (2023) found that, in recycling Acrylonitrile Butadiene Styrene (ABS) and High Impact Polystyrene (HIPS) from WEEE, extrusion consumed less energy (0.7 kWh/kg) compared to injection molding (2.0 kWh/kg), which also generated more solid waste. Garcia et al. (2021) compared the environmental impacts and mechanical properties of conventional manufacturing (injection molding) and additive manufacturing (FDM) using recycled ABS, concluding that additive manufacturing is more sustainable for small batches, while injection molding is more efficient for larger ones.

At a broader scale, Ardolino et al. (2021) applied LCA to evaluate the environmental sustainability of European WEEE plastic management. The methodology considered different management scenarios, including mechanical recycling, energy recovery, and improper disposal, such as open burning and dumping. The results showed that mechanical recycling and energy recovery are significantly more sustainable than improper management options. Scenarios that involve exporting waste to non-European countries, where they are often managed under substandard conditions, resulted in substantially higher environmental impacts, particularly in terms of carcinogenic potential and terrestrial ecotoxicity.

Similarly, Rocha and Penteado (2021) and Sun et al. (2024) reinforced the environmental advantages of recycling WEEE plastics in different regional contexts. Among these studies, none have applied Life Cycle Impact Assessment (LCIA) methods combined with a comprehensive accounting of both benefits and burdens from reverse logistics and the recycling of HIPS and ABS derived from WEEE in the Brazilian context. Furthermore, detailed impact data for these processes remain limited, highlighting the need for studies that contribute to national databases and support decision-making in this area.

This study aims to assess the environmental impacts of a WEEE plastic recycling process, focusing on ABS and HIPS, the main polymers present in WEEE, through a case study conducted at a WEEE plastic recycling company located in Sorocaba, São Paulo, Brazil, using LCA. This company is one of the leading recyclers in the country, responsible for reverse logistics operations, recycling, and the proper disposal of components not treated internally. It collects WEEE from across Brazil. Aligned with the principles of the circular economy, the company operates across multiple stages, from planning and executing reverse logistics, disassembly, and destruction of post-consumer electronic devices, to the development and production of new materials and plastic components. Additionally, this study provides detailed impact data for these processes, contributing valuable information to Brazilian databases and advancing the understanding of sustainable WEEE management practices in the region.

## Materials and methods

**Fig. 1:**
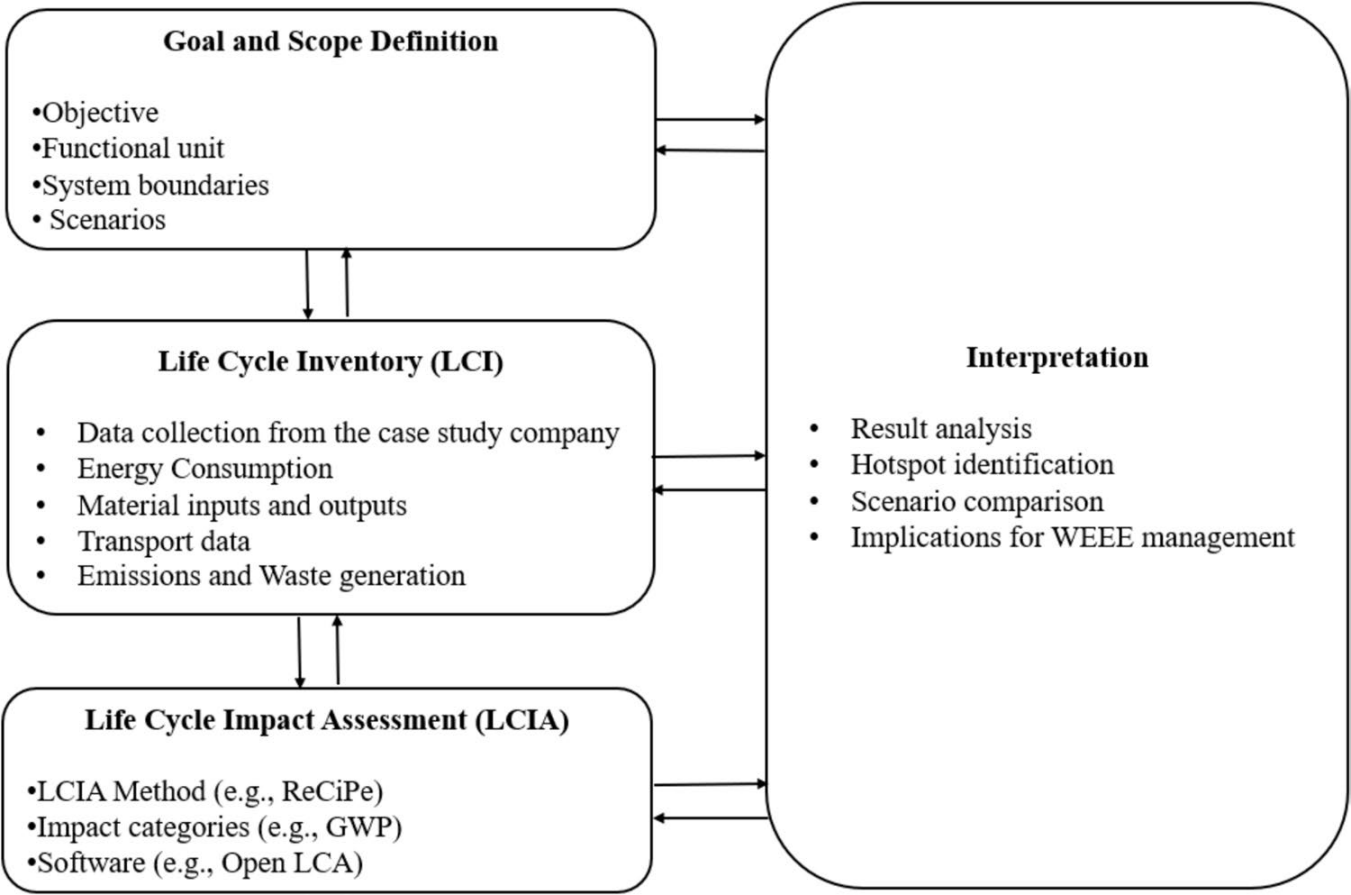
Framework of the Life Cycle Assessment (LCA) applied in this study. The figure presents the four main phases of LCA—goal and scope definition, life cycle inventory (LCI), life cycle impact assessment (LCIA), and interpretation—along with the specific tasks performed in each stage

The LCA study was conducted according to the guidelines of ISO 14040 (International Organization for Standardization 2006a) and ISO 14044 (International Organization for Standardization 2006b) standards. The data and assumptions of the study are detailed in the following sections. Figure 1 presents the methodological framework of the LCA conducted in this study, illustrating the standard phases and the main tasks performed in each stage.

### Definition of goal and scope

The goal of this LCA is to examine the environmental impacts of producing HIPS and ABS from the recycling of WEEE plastics. This study aims to identify the hotspots of the recycling process at a company located in Sorocaba, SP.

The system boundary defined for this analysis is “gate-to-gate” (from WEEE collection and transportation to the production and storage of recycled ABS/HIPS pellets), encompassing all stages from the input of waste into the recycling company to the production of recycled pellets. The product system under study includes all operations performed by the WEEE recycling company, from waste collection to the storage of recycled pellets.

The geographical coverage of the study is limited to the recycling plant located in Sorocaba, SP. The function of the product system is to produce recycled HIPS and ABS pellets, which can be used as plastic raw materials by various industries, considering the reintegration of these materials into the production chain, reinforcing the significance of the circular economy. For this analysis, the functional unit was defined as 1 kg of HIPS and 1 kg of ABS.

The reference flow to produce 1 kg of HIPS requires 1.075 kg of ground HIPS extracted from WEEE, and to produce 1 kg of ABS, 1.0944 kg of ground ABS extracted from WEEE is needed. The allocation of environmental impacts will be based on the mass of the processed materials. The mass of plastics present in WEEE is approximately 19.6%, and the selected allocation procedure was the mass method.

The subsystems considered in the analysis include the stages of WEEE collection, waste transportation, material shredding, component separation, pellet extrusion, and product storage. The data collected for the analysis includes primary information obtained directly from the company in 2023, which was used to update an inventory from literature (Mendes Campolina et al. 2017) and secondary data extracted from LCA databases (Ecoinvent 3.10) and literature.

The recycled HIPS and ABS pellets produced by the company in Sorocaba, SP, are reintroduced as plastic raw materials for various industries, contributing to the circular economy and reducing the environmental impacts associated with the production of virgin plastics.

### Process map

The studied company follows a process like those outlined in the literature. According to Mendes Campolina et al. (2017), the process begins with the collection and transportation of Waste Electrical and Electronic Equipment (WEEE) to the facility. Upon arrival, the WEEE undergoes a separation process, where different components are sorted, including plastics, ferrous metals, PCBs, HDDs, SSDs, cables, solid waste (e.g., packaging), and others. This disassembly is typically performed manually. Subsequently, plastics are identified and sent to the shredder, while co-products such as PCBs and other materials are forwarded to specialized companies for recycling. During the shredding process, solid residues are also removed. Finally, plastics are identified and separated precisely using near-infrared (NIR) spectroscopy and pyrolysis (Py-IR) technologies (Nanda and Berruti 2021; Xia et al. 2021). The shredded plastics are then processed through extrusion, producing pellets. The pellets are stored until they are sold.

Non-ferrous metals, aluminum, and plastics are separated using techniques such as magnetism and density separation (Tutton et al. 2022). The separated materials are sent for different treatments: ferrous metals to steel mills, plastics for specific recycling, and copper and aluminum to foundries. Materials considered rejects can be sent to landfills (Araújo et al. 2012).

**Fig. 2:**
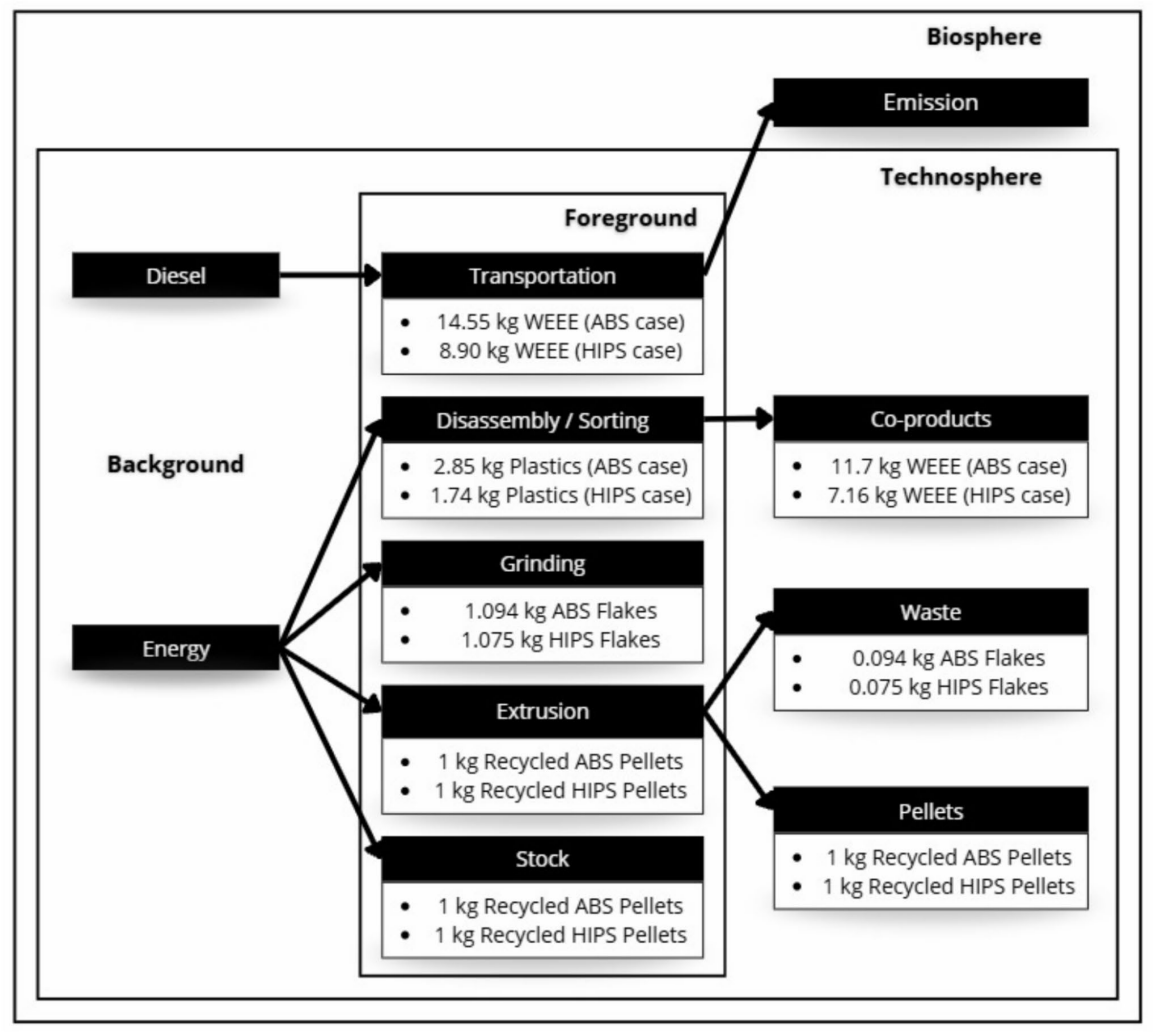
Studied system: WEEE recycling process

Figure 2 illustrates the studied system, outlining the inputs, outputs, and the WEEE recycling process. The diagram simplifies the process steps described, including transportation, dismantling/separation, grinding, extrusion, and storage (foreground data). Additionally, it identifies the generation of waste and co-products as well as the energy sources used and associated emissions (background data). This representation provides an overview of the interactions between the technosphere and the biosphere within the analyzed system.

### Life cycle inventory (LCI)

The LCIs were developed using the study by Mendes Campolina et al. (2017) as a foundational basis. However, to ensure the inventory reflects current industrial practices, these data were updated and supplemented with primary data collected in 2023 from a company specializing in recycling plastics from WEEE.

The selected recycling facility is representative of the Brazilian WEEE plastics recycling sector, as it operates under typical technological conditions observed in the country, relying on mechanical recycling routes (manual dismantling, shredding, NIR/Py-IR sorting and extrusion), without advanced chemical recycling or fully automated separation systems. These characteristics reflect the current average technological maturity of WEEE plastic recycling in Brazil.

#### LCI of the ABS and HIPS recycling process

Table 1 presents the LCI of the ABS and HIPS recycling process and the flows and providers from Ecoinvent chosen in the OpenLCA 2.1.1 software for modeling.

**Table 1:** LCI of ABS and HIPS recycling process

Parameter	Unit	Quantity (ABS)	Quantity (HIPS)	Flow Connection with Ecoinvent Database
Diesel for WEEE transportation	kg/kg of recycled ABS/HIPS	0.05152	0.08421	*Diesel | market for diesel | diesel | Cut-off, U—BR*
Carbon dioxide from transportation		0.2226	0.1362	*Carbon dioxide, fossil*
dinitrogen monoxide from transportation		0.00001795	0.000010984	*Dinitrogen monoxide*
Methane from transportation		0.00001795	0.000010984	*Methane, fossil*
Carbon dioxide, non-fossil from transportatio		0.03231	0.01977	*Carbon dioxide, non-fossil*
WEEE arriving at the company		14.5542	8.9042	*electronics scrap*
WEEE sent to the disassembly and separation area		14.5542	8.9042	*electronics scrap*
WEEE plastics in disassembly and separation area		2.8517	1.7446	*Waste plastic, consumer electronics, unsorted*
Other WEEE components		11.7025	7.1595	*electronics scrap*
WEEE-derived ABS/HIPS plastics to be ground		1.0944	1.07506	*Waste plastic, consumer electronics, sorted*
Ground WEEE-derived ABS/HIPS plastics to be extruded		1.0944	1.07506	*Plastic flake, consumer electronics, for recycling*
ABS/HIPS pellets		1.0000	1.0000	*acrylonitrile–butadiene–styrene copolymer (ABS), polystyrene, high impact (HIPS)*
Extrusion waste		0.0944	0.07506	*waste plastic, consumer electronics*
Waste treatment		0.95	0.58	*process-specific burdens, municipal waste incineration | Cut-off, U—RoW*
Energy – disassembly and separation	kWh/kg of recycled ABS/HIPS	0.65	0.4	*electricity, medium voltage, Cut-off, U—BR—South-eastern/Mid-western grid*
Energy—grinding		0.28	0.14	*electricity, medium voltage, Cut-off, U—BR—South-eastern/Mid-western grid*
Energy—extrusion		1.92	2.14	*electricity, medium voltage, Cut-off, U—BR—South-eastern/Mid-western grid (normal energy)/* *market for electricity, medium voltage, renewable energy products | electricity, medium voltage, renewable energy products | Cut-off, U – CH (hypothetical renewable energy scenario)*
Energy—stock		0.76	0.47	*electricity, medium voltage, Cut-off, U—BR—South-eastern/Mid-western grid*

This detailed inventory forms the basis for the LCA, providing data on the environmental impacts associated with each step of the ABS and HIPS recycling process. The inputs and outputs, including energy consumption, emissions, and waste generation, are necessary for modeling the environmental performance of the recycling processes using the Open LCA with Ecoinvent data.

Primary foreground data were collected at the recycling facility in 2023 and represent typical operating conditions observed during the data collection period. Where applicable, operational data were compiled as averaged values over the available monitoring period, and the potential influence of seasonal variability is discussed as a limitation.

The extrusion process operates under a closed-loop water system, in which process water is continuously recirculated for cooling purposes. No wastewater is discharged outside the facility, and no external wastewater treatment is required. Therefore, no effluent flows were included in the LCI, and water use is accounted for exclusively as recirculated utility water within the system boundary. Moreover, the extrusion process energy consumption includes not only the extruder itself but also auxiliary systems such as material suction pumps, lubrication pumps, pigment and additive pumps, cooling tower pumps, and the pelletizer. While these systems are integral to the extrusion line, the energy consumption data for extrusion in the present study reflect the total energy use of the entire extrusion system.

Solid residues generated during dismantling and sorting correspond mainly to WEEE fractions not treated within the studied facility (e.g., specific components or materials forwarded to specialized downstream operators). As these flows fall outside the organizational and technological control of the recycling plant and are not functionally related to the production of recycled ABS/HIPS pellets, they were excluded from the system boundary, in accordance with ISO 14044 cut-off criteria. Their management is therefore not attributed to the functional unit assessed in this study.

Background datasets were sourced from ecoinvent v3.10, cut-off system model. Therefore, multifunctionality in background processes follows the cut-off conventions, and recycling credits are addressed through the applied approach (CFF/avoided burden) rather than background substitution modeling.

### Impact assessment

The impact categories and LCA methods were selected based on the recommendations of International Organization for Standardization (ISO, 2006b). Therefore, the ReCiPe method (ReCiPe 2016 v1.03, midpoint (H)), used in the OpenLCA served as the calculation basis.

### Interpretation

For the sensitivity analysis, several scenarios were selected: (i) reduction in the use of electricity in extrusion (−10%, −20%, and −30% compared to the baseline), increase in the transportation distance of WEEE and change in the energy source to the renewable energy mix (electric, medium voltage, renewable energy products (Cut-off, U—BR—South-eastern/Mid-western grid)), as well as changes in the allocation criteria. The average distance traveled for the collection and transportation of all WEEE was 3089 km, equivalent to 0.08 km/kg of recycled HIPS and 0.14 km/kg of recycled ABS after being converted to the functional unit and allocated to the plastics within the WEEE, as described by Mendes Campolina et al. (2017). Based on this baseline, successive increases in this distance were applied, resulting in monthly transportation distances of 6179 km, 6767 km, 7414 km, 8032 km, and 8650 km (100%, 119%, 140%, 160%, 180%). A sensitivity analysis was also conducted by changing the allocation method to economic allocation, which resulted in variations in the impacts, as other components of WEEE, such as metals, have a higher economic value compared to plastics.

Economic allocation was applied to distribute the foreground burdens among the output streams generated during WEEE dismantling and sorting. Allocation factors were calculated at the material level, based on the economic value of each output stream, defined as the product of mass share and unit price, considering the average material composition of WEEE processed by the company.

Unit prices were collected from multiple online sources and reflect current market conditions (European Environment Agency 2024; Metaloop 2025; Business Analytiq 2025; Paz Metals 2025), and two alternative allocation scenarios were evaluated to address price variability and market uncertainty. The first scenario adopts scrap-based prices, reflecting typical values for dismantled materials traded as secondary raw materials, expressed in EUR per tonne. The second scenario adopts commodity-based prices, reflecting international commodity market values, expressed in USD per tonne. Each scenario was calculated independently using a single currency.

Prices refer to post-sorting material output streams, at the plant gate or point of sale. In the studied system, all material fractions are valorized and forwarded to recycling or recovery routes, and therefore no disposal to landfill or co-processing occurs. Material fractions previously grouped as residual streams were reassigned and redistributed among the valorized material outputs, and mass shares were renormalized prior to the allocation calculation.

A transparent calculation table, including mass shares, unit prices, data sources, and resulting allocation factors, is provided in Table S1 (Supplementary Material). The comparison between scrap-based and commodity-based allocation factors allows assessment of the sensitivity of the results to the choice of economic valuation approach, as recommended in LCA studies involving WEEE and secondary materials.

For PCBs, the commodity-based price was derived from the PCB metal composition and corresponding metal prices (Table S2).

The baseline electricity supply reflects the Brazilian South-eastern/Mid-western interconnected grid, consistent with the geographical location and utility supply of the studied facility. The 100% renewable electricity scenario represents a hypothetical exploratory scenario, designed to evaluate the potential environmental benefits of a fully decarbonized electricity supply. This scenario does not correspond to a specific commercial electricity product currently used by the company and should therefore be interpreted as a long-term, not intended to represent current policy or market conditions.

### Comparison with virgin ABS and HIPS production

Considering that recycling is a multifunctional operation, the environmental burdens and benefits associated with the recycling of ABS and HIPS from WEEE were assessed using the Circular Footprint Formula (CFF), as in the PEF Guide (European Union Joint Research Centre (EU-JRC) 2018). The CFF approach was applied to allocate impacts between the primary and secondary material cycles.

The allocation factor A was set to 0.5, in accordance with the range recommended by the PEF guidance (0.2–0.8). This value reflects a balanced attribution of environmental burdens and benefits between recycled content and end-of-life recycling, which is particularly appropriate for ABS and HIPS derived from WEEE in the Brazilian context. The Brazilian WEEE plastics market is characterized by growing but still limited demand for recycled polymers, alongside regulatory pressure to increase recycling rates. Therefore, assigning equal importance to recyclability at end-of-life and the use of recycled content avoids overemphasizing either upstream or downstream benefits and represents a conservative, context-appropriate choice.

The recycling efficiency parameter (*R*2) was calculated from primary data based on the actual material flows entering and exiting the recycling process. The *R*2 value represents the ratio of recycled ABS/HIPS pellets produced relative to the plastic fractions entering the process. The calculated *R*2 values were 93% for HIPS and 90% for ABS, reflecting the material recovery efficiency of the recycling facility under real industrial operating conditions. These high recovery rates confirm the effectiveness of the recycling process in the plant, accounting for losses during dismantling, shredding, sorting, and extrusion stages.

The quality ratio between secondary and primary materials (Qsout/Qp) was quantified based on experimentally measured mechanical properties reported by Hirayama and Saron (2018). Ratios between recycled and virgin polymers were calculated for tensile strength, Young’s modulus, and impact resistance, using the data presented in Table 2 of the cited study.

**Table 2:** Impact category results for ABS and HIPS

Results			
		**HIPS**	**ABS**
**Impact Category**	**Unit**	**Result**	**Result**
Acidification: terrestrial—terrestrial acidification potential (TAP)	kg SO2-Eq	1.320E-03	1.350E-03
Climate change—global warming potential (GWP100)	kg CO2-Eq	5.888E-01	6.161E-01
Ecotoxicity: freshwater—freshwater ecotoxicity potential (FETP)	kg 1,4-DCB-Eq	1.445E-02	1.463E-02
Ecotoxicity: marine—marine ecotoxicity potential (METP)	kg 1,4-DCB-Eq	2.428E-02	2.461E-02
Ecotoxicity: terrestrial—terrestrial ecotoxicity potential (TETP)	kg 1,4-DCB-Eq	6.867E + 00	6.971E + 00
Energy resources: non-renewable, fossil—fossil fuel potential (FFP)	kg oil-Eq	1.415E-01	1.502E-01
Eutrophication: freshwater—freshwater eutrophication potential (FEP)	kg P-Eq	3.184E-05	3.231E-05
Eutrophication: marine—marine eutrophication potential (MEP)	kg N-Eq	3.898E-05	3.974E-05
Human toxicity: carcinogenic—human toxicity potential (HTPc)	kg 1,4-DCB-Eq	4.583E-02	4.676E-02
Human toxicity: non-carcinogenic—human toxicity potential (HTPnc)	kg 1,4-DCB-Eq	2.782E-01	2.817E-01
Ionising radiation—ionising radiation potential (IRP)	kBq Co-60-Eq	8.071E-02	8.149E-02
Land use—agricultural land occupation (LOP)	m2*a crop-Eq	4.359E-02	4.404E-02
Material resources: metals/minerals—surplus ore potential (SOP)	kg Cu-Eq	7.440E-03	7.580E-03
Ozone depletion—ozone depletion potential (odpinfinite)	kg CFC-11-Eq	1.696E-06	1.729E-06
Particulate matter formation—particulate matter formation potential (PMFP)	kg PM2.5-Eq	7.800E-04	7.900E-04
Photochemical oxidant formation: human health—photochemical oxidant formation potential: humans (HOFP)	kg NOx-Eq	1.210E-03	1.260E-03
Photochemical oxidant formation: terrestrial ecosystems—photochemical oxidant formation potential: ecosystems (EOFP)	kg NOx-Eq	1.270E-03	1.320E-03
water use—water consumption potential (WCP)	m3	4.491E-02	4.537E-02

For HIPS, the average ratio was approximately 1.0, indicating functional equivalence between recycled and virgin material. For ABS, due to a significant reduction in impact resistance associated with degradation of the rubber phase, the calculated average ratio was approximately 0.8, reflecting partial downcycling effects. These values were directly adopted as Qsout/Qp in the Circular Footprint Formula implementation.

The equation utilized is:

$$E = \left(1 - A\right){R}_{2}\cdot \left.\left( {E}_{\left\{recyclingEoL\right\}}- {E}_{v}^{*}\frac{{Q}_{sout}}{Qp}\right.\right)$$

where:

- **A:** Allocation factor for credits and impacts between the supplier and user of the recycled material. $0.2\le A\le 0.8.$.
- **R**_**2**_**​:** Proportion of material in the product that will be recycled. The *R*2​ factor must consider inefficiencies in the recycling process and should be measured at the output of the recycling plant.
- **E**_**recycling EoL**_**​:** Impact of the recycling process, including collection, sorting, and recycling operations.
- ${{\boldsymbol{E}}}_{{\boldsymbol{v}}}^{\boldsymbol{*}}$**:** Impact of virgin material assumed to be substituted (avoided).
- ${{\boldsymbol{Q}}}_{{\boldsymbol{s}}{\boldsymbol{o}}{\boldsymbol{u}}{\boldsymbol{t}}}$**​:** Quality of the secondary material at the substitution point.
- ${\boldsymbol{Q}}{\boldsymbol{p}}$ **​:** Quality of the primary (virgin) material.

Additionally, the calculation of these credits was also performed using only the avoided burden approach, which involves subtracting the environmental impact of virgin plastic production from the calculated impact of the recycling operation, to compare it with the CFF approach.

Virgin material substitution was modeled using Ecoinvent v3.10 Cut-off datasets “polystyrene production, high impact | polystyrene, high impact | RoW (Rest of World)” and “acrylonitrile–butadiene–styrene copolymer production | ABS | RoW”. These datasets represent primary polymer production at comparable material grades (HIPS and ABS). A RoW geographical scope was adopted to represent international supply conditions for polymers imported to Brazil; therefore, the embedded electricity and energy mixes correspond to global production averages rather than a Brazilian-specific grid.

### Uncertainty analysis

For the uncertainty analysis, data evaluation was conducted using the Pedigree Matrix as described by Weidema et al. (2013), which quantifies the inherent uncertainty of the study's data. This matrix evaluates data quality based on five criteria: reliability, completeness, temporal correlation, geographical correlation, and technological correlation.

## Results and discussion

### LCA results

After performing the LCIA using the ReCiPe 2016, midpoint (H) method, the environmental impacts of the recovery processes for ABS and HIPS were analyzed. The extrusion stage emerged as the most impactful in both cases, accounting for approximately 75.2% of the total environmental impacts for ABS and about 85.7% for HIPS. This result is mainly explained by the high electricity demand associated with the extrusion process, which dominates the overall environmental profile of the system.

This result is partially consistent with previous studies on WEEE plastic recycling. For instance, recent research indicates that disassembly and granulation processes can account for more than 90% of total environmental impacts, with electricity consumption as the main driver (Spirio et al. 2024). While these studies refer to broader pre-processing stages, the present study identifies extrusion as the dominant contributor, which can be interpreted as a more granular identification of the energy-intensive step within mechanical recycling. Similarly, energy consumption has been identified as the dominant contributor across multiple impact categories, often exceeding 70% of total impacts, while transportation plays a comparatively smaller role (Sun et al. 2024). These findings support the results obtained in this study, particularly regarding the central role of electricity use in shaping environmental performance.

In the Brazilian context, Rocha and Penteado (2021) also highlighted the environmental benefits of proper WEEE management, although their results emphasized the overall advantages of recycling systems rather than the contribution of individual process stages. This reinforces the importance of studies such as the present one, which provide a more detailed breakdown of environmental hotspots within recycling operations.

**Fig. 3:**
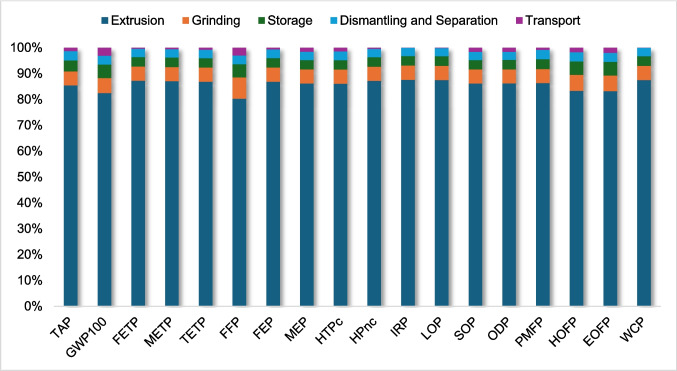
Contribution analysis (HIPS)

**Fig. 4:**
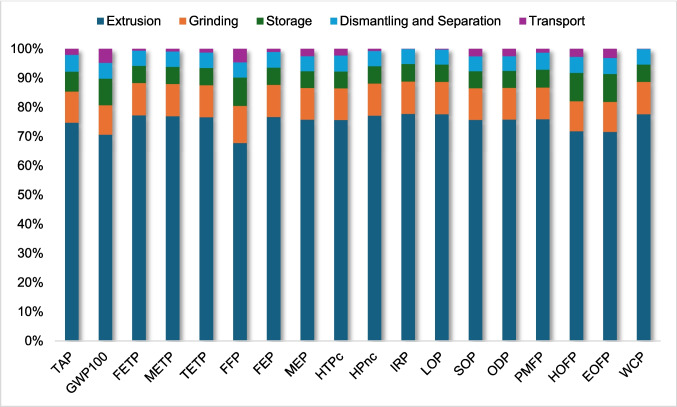
Contribution analysis (ABS)

It is important to note that these results may vary if the analysis is conducted in another country, because electricity consumption was modeled using ecoinvent’s Brazilian grid dataset, in which hydropower accounts for a significant share, alongside smaller contributions from thermal, nuclear, and other energy sources. In contrast, transportation contributed comparatively less to the overall environmental impacts, reinforcing the dominant role of electricity consumption in the system. Table 2 presents the results obtained for both plastics, while Figs. 3 and 4 show the contribution of each stage of the process to the final result for HIPS and ABS, respectively.

The contribution analysis further shows that grinding is the second most impactful stage, followed by storage. Although these stages also consume electricity, their contribution is considerably lower than that of extrusion, indicating that the intensity and duration of energy use in extrusion are the main drivers of environmental impacts. This lower contribution of transportation can be attributed to the relatively low fuel consumption per functional unit and the predominance of electricity-related impacts in the system.

Furthermore, the dominance of the extrusion stage can also be explained by its intrinsic process characteristics. Experimental studies have shown that extrusion of recycled ABS and HIPS can require between approximately 2.5 and 7 MJ/kg, depending on process conditions and material characteristics (Garcia et al. 2021). This high energy demand directly translates into increased environmental impacts, particularly in impact categories sensitive to energy use, such as climate change, resource depletion, and toxicity-related categories.

The relatively lower contribution of transportation observed in this study is also aligned with the literature. Although transportation can be relevant in certain impact categories such as acidification and eutrophication, its overall contribution remains limited when compared to electricity consumption in recycling processes (Spirio et al. 2024). This behavior is particularly expected in systems where most operations are centralized, reducing transport distances and associated emissions.

**Fig. 5:**
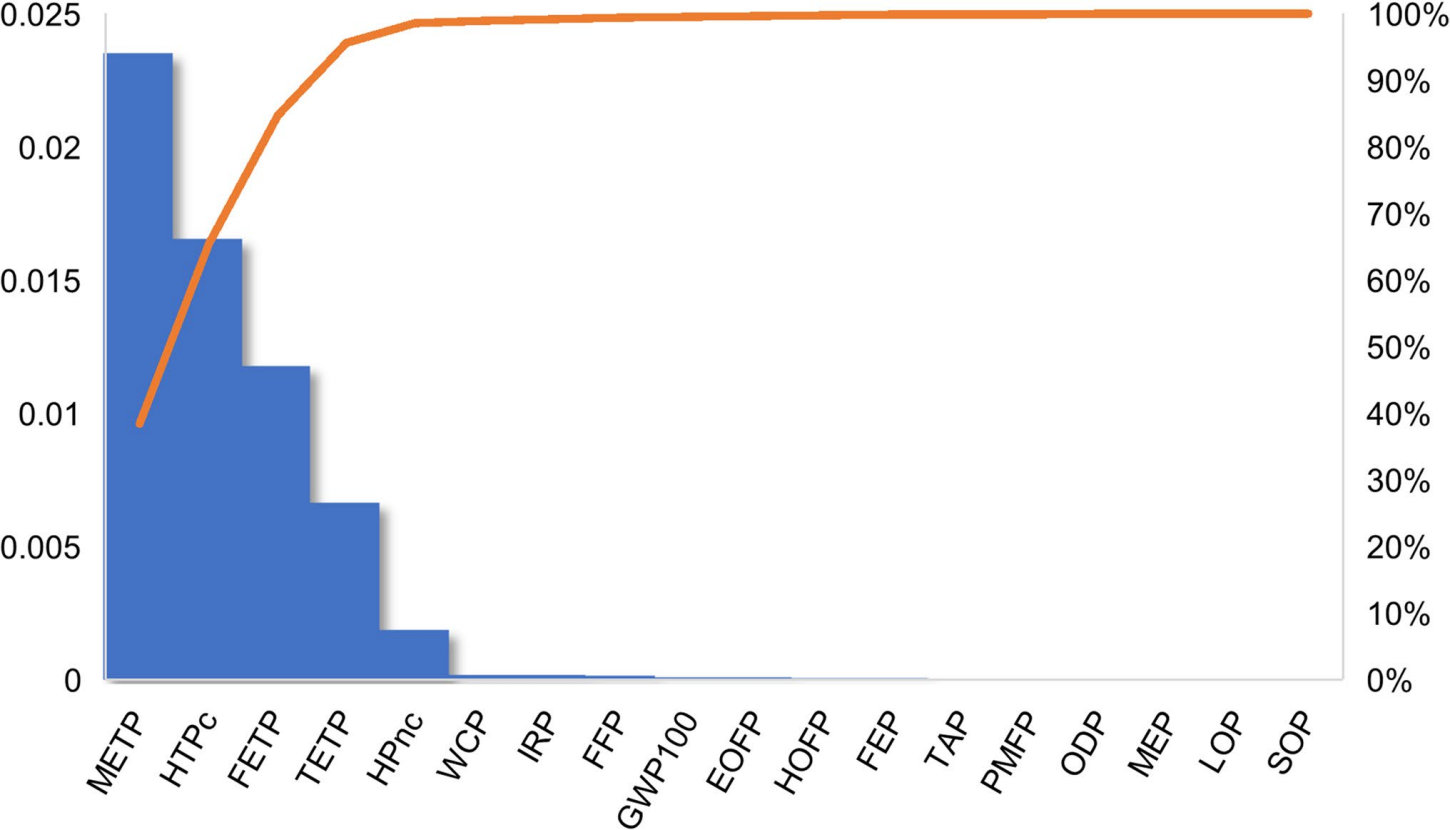
Normalized environmental impacts by category for HIPS recycling process (% contribution)

**Fig. 6:**
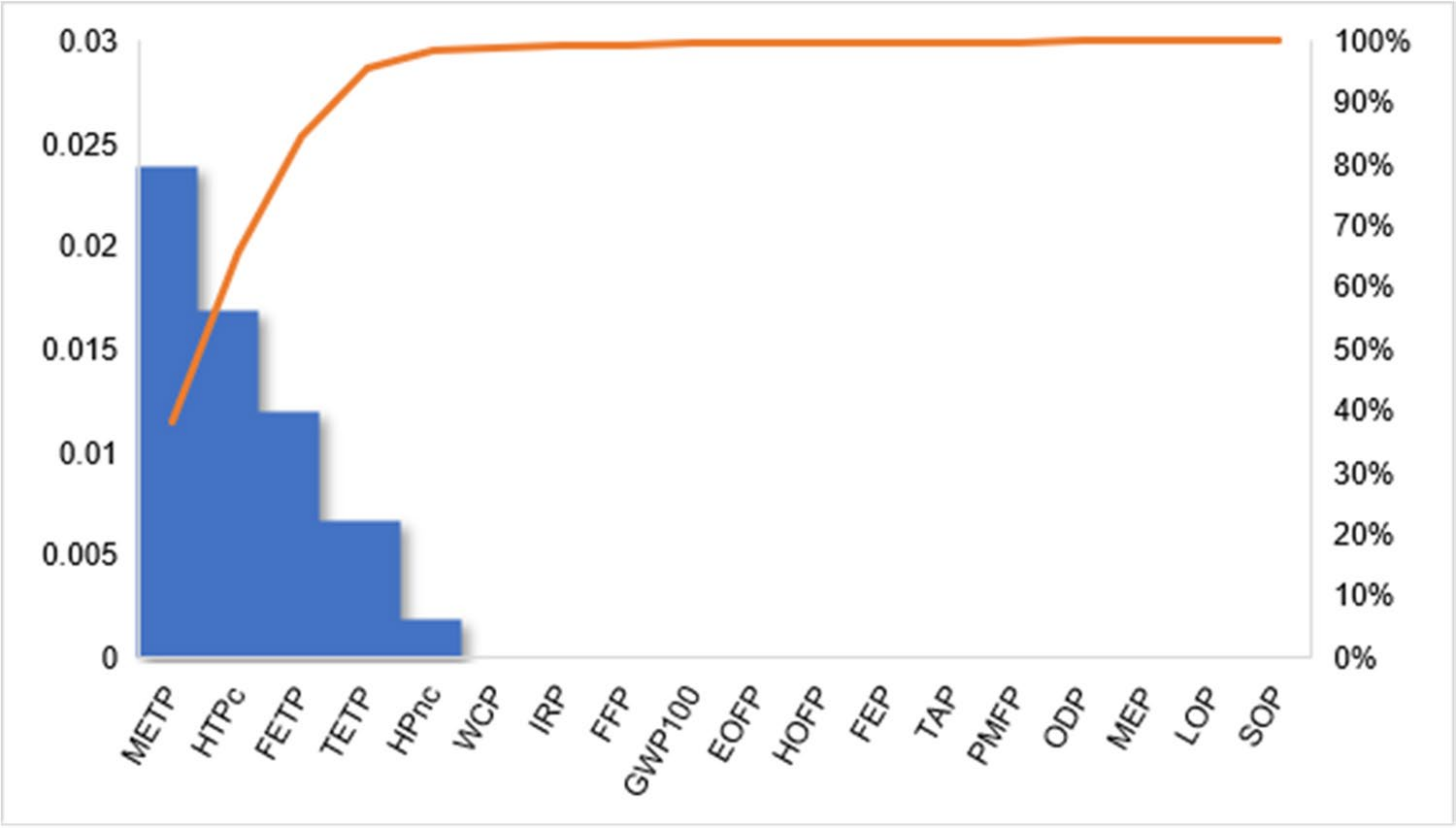
Normalized environmental impacts by category for ABS recycling process (% contribution)

Beyond process-level contributions, the most relevant impact categories were identified through normalization using the World H/H 2010 factors from the ReCiPe 2016 method. The results indicate that, for both plastics, the main categories are marine ecotoxicity potential, human toxicity potential (cancer), freshwater ecotoxicity potential, terrestrial ecotoxicity potential, human toxicity potential (non-carcinogenic), water consumption, ionizing radiation potential, fossil resource depletion, and global warming potential. These categories account for up to 99.6% of the total impacts. The remaining impacts are distributed across the other eight categories. Figures 5 and 6 present diagrams showing the normalized impacts of each category and their percentage contributions for HIPS and ABS, respectively.

This profile is also consistent with previous LCA studies on WEEE recycling, which report a strong contribution of ecotoxicity-related categories and human toxicity due to emissions associated with electricity generation and material processing (Spirio et al. 2024; Sun et al. 2024). Additionally, the relevance of resource depletion and climate change categories further highlights the importance of energy consumption as a key environmental driver in recycling systems.

Overall, the results demonstrate that improving the energy efficiency of extrusion and other mechanical processing stages represents the most effective strategy for reducing environmental impacts in WEEE plastic recycling. This highlights electricity consumption as the critical leverage point for environmental optimization, reinforcing its central role in circular economy strategies for WEEE management.

### Sensitivity analysis

To analyze the potential changes in environmental impacts resulting from alterations in the system studied, several scenarios were established and evaluated. Given that the primary hotspot of the system is extrusion due to its high energy consumption, scenarios involving a reduction in energy use by 10%, 20%, and 30% were considered. These reductions could be practically achieved by implementing energy-efficient machinery or optimizing process parameters, such as temperature and throughput, during extrusion.

Conversely, as transportation had a relatively low impact, increases in the transportation by 100%, 119%, 140%, 160%, and 180% were evaluated, reflecting potential changes in logistics networks or expanded collection areas. The high rates of increase were justified by the low relevance of transportation impacts in the system's overall profile. Additionally, a scenario considering the exclusive use of renewable energy sources was analyzed, which could be practically integrated by sourcing electricity from renewable grids or installing on-site renewable energy systems, such as solar panels, at recycling facilities.

**Fig. 7:**
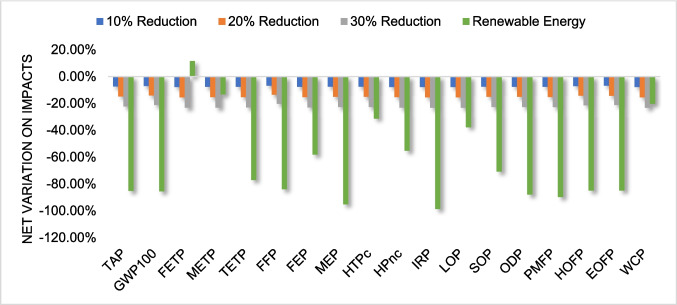
Percentage variation in major impacts by changing energy use during extrusion for ABS

**Fig. 8:**
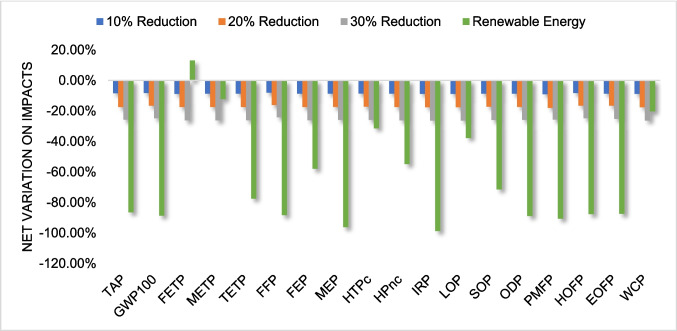
Percentage variation in major impacts by changing energy use during extrusion for HIPS

Figures 7 and 8 show, respectively, the percentage variation in impact category results due to the reduction in energy use during extrusion for ABS and HIPS and the exclusive use of renewable energy sources.

From the analysis of the graphs, it is possible to observe that, proportional to the reduction in energy use, there was a considerable decrease in environmental impact across all analyzed categories, of the same magnitude. This indicates that reducing energy consumption, through the optimization of heat exchangers and other elements of the extrusion process, can be an effective strategy to improve LCA indicators.

With the use of 100% renewable energy in all processes, the results show a significant reduction in impacts, highlighting the importance of integrating renewable energy sources as a strategic approach to minimize negative environmental effects, as noted by Rabbi et al. (2022). These results suggest that impact decreases significantly in this scenario. However, changing the energy mix is more complex as it involves a structural change implemented in the long term. Despite this complexity, the results provide an important insight into the importance of replacing fossil energies with renewable sources.

**Fig. 9:**
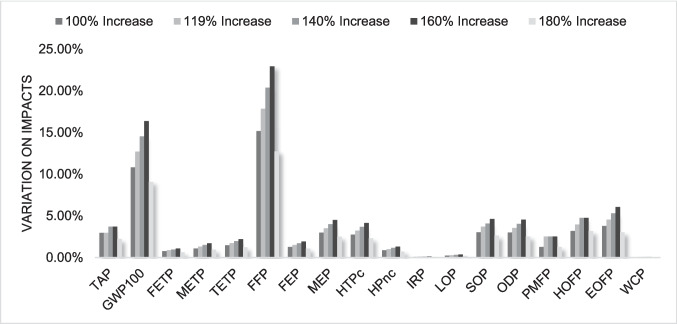
Percentage variation in major impacts by increasing transportation distance for ABS

**Fig. 10:**
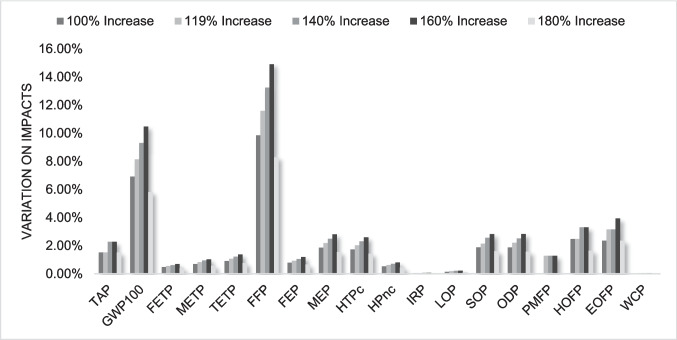
Percentage variation in major impacts by increasing transportation distance for HIPS

Regarding transportation, despite substantial changes in the analyzed parameters, there was no significant increase in the system's environmental impacts, as shown in Figs. 9 and 10. The categories with the most significant growth are those related to human toxicity and terrestrial ecotoxicity potential. This confirms that transportation is not a hotspot in the studied case, aligning with Rocha and Penteado (2021) findings, which demonstrated that only very substantial changes in transportation parameters lead to a significant increase in system impacts and Sun et al. (2024) that identified electricity consumption as the primary hotspot, surpassing transportation in terms of environmental impact, even though their study only extended to the granulation stage. Similarly, this finding aligns with the present study, where electricity was also the dominant contributor.

**Fig. 11:**
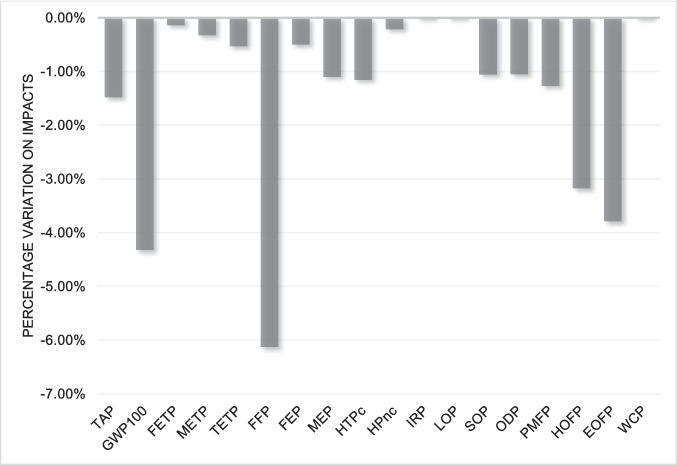
Comparison between mass allocation and economic allocation for ABS

**Fig. 12:**
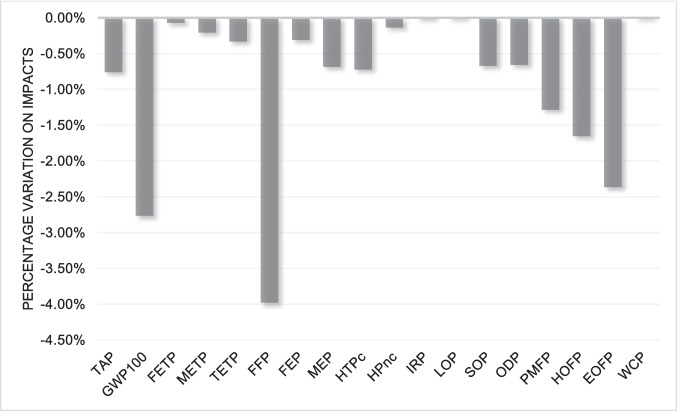
Comparison between mass allocation and economic allocation for HIPS

Finally, changing the allocation criteria can also bring significant changes to the LCIA results. Given that the initial criterion used was mass-based, a new version considering the economic criterion was considered. From this analysis, the allocation factor for WEEE-derived plastics was determined to be 10%, reflecting the economic value of the plastics relative to all materials present in WEEE, reflecting the relative economic value of plastics compared to other WEEE fractions, which are typically dominated by metals (Pokhrel et al. 2020). The results show a drop in the impacts. This is because the highest value present in WEEE is in the metal fraction, as stated by Pokhrel et al. (2020). The economic data for WEEE and its components were obtained from a combination of the Ecoinvent database, market sources and literature. Figures 11 and 12 show the percentage variation concerning the result with mass allocation for ABS and HIPS, respectively.

### Comparison with virgin ABS and HIPS production

**Fig. 13:**
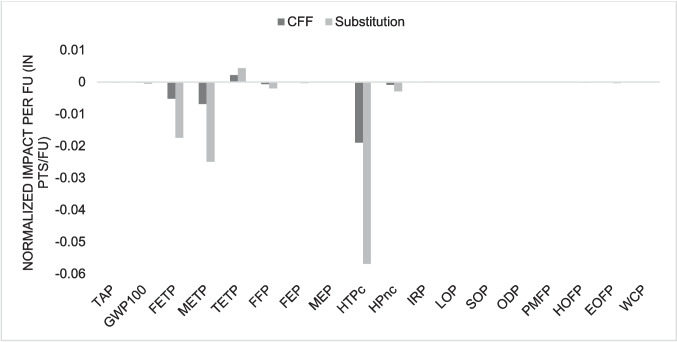
Comparison between recycled ABS production and virgin ABS production

**Fig. 14:**
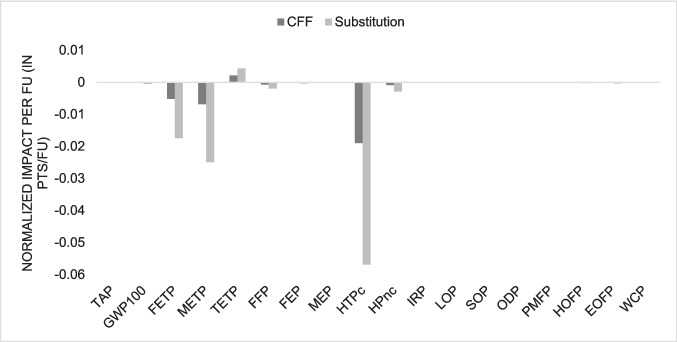
Comparison between recycled HIPS production and virgin HIPS production

Figures 13 and 14 present graphs displaying the results of subtracting the impacts of virgin and recycled plastic production for each considered impact category, comparing calculations using the CFF and the simple substitution approach. Despite some categories showing almost equal results for virgin and recycled plastic, the data analysis reveals that recycled plastic exhibits considerably better environmental performance than virgin plastic in certain categories. Negative values indicate environmental benefits of recycling compared to virgin plastic production.

The most notable improvement is observed in the global warming category, which shows the most negative value for both ABS and HIPS. These findings suggest that plastic recycling should be encouraged to allow materials to remain in the technosphere for as long as possible, thereby avoiding the extraction of virgin raw materials to produce new plastics.

Similarly, Teixeira et al. (2023) noted that extrusion, as a process, results in lower environmental impacts compared to the production of virgin plastics, further reinforcing the idea that recycling WEEE plastics represents a more sustainable alternative to conventional plastic production. Sun et al. (2024) also emphasizes the significant role of WEEE plastic recycling in mitigating environmental impacts, highlighting that these benefits could be amplified with the adoption of more advanced and efficient recycling technologies.

The use of the CFF is particularly interesting due to the inclusion of additional parameters in the equation, such as recycling efficiency, a market demand factor, and a quality factor for recycled material. These factors account for scenarios where recycled material alone may not entirely replace the same amount of virgin material, often requiring the addition of other materials to achieve properties equivalent to virgin plastics. Therefore, the CFF provides a more precise evaluation for materials like plastics, reflecting their unique characteristics and the complexities of their recycling processes.

### Uncertainty analysis

Data quality was assessed using a simplified pedigree matrix approach, following established LCA practice. Five dimensions were evaluated: reliability, completeness, temporal correlation, geographical correlation, and technological correlation. Primary data collected directly at the company were assigned high-quality scores, reflecting direct measurements, strong representativeness of the studied system, and recent data collection.

The aggregated pedigree scores are presented in Table 3 and indicate a high level of confidence in the foreground inventory data used in this study. In addition, a detailed flow-level pedigree matrix, including individual scores and justifications for each inventory flow, is provided in the Supplementary Information (Table S3). This detailed assessment enhances transparency and allows for a more comprehensive evaluation of data quality and associated uncertainties.

**Table 3:** Pedigree matrix scores

Aspect	Score	Justification
Reliability	1	Data measured directly at the company and, if possible, verified through audits or internal validations
Completeness	2	Even though it is a single company, if the energy and transport data were collected over an entire year or during different operational periods, it adequately covers variability
Temporal correlation	1	The data were collected in 2023
Geographical correlation	1	The data are directly from the specific location of the study, ensuring high geographical representativeness
technological correlation	1	Data obtained directly from the company's processes and technologies are highly representative

Overall, the results show that most foreground data are based on primary measurements (reliability score = 1), while some parameters—such as transport-related emissions and energy consumption—were estimated using established methodologies or secondary data sources, resulting in slightly higher uncertainty (reliability score = 2). Despite these limitations, the combination of high representativeness and consistent data collection supports the robustness of the life cycle inventory and the reliability of the impact assessment results.

## Conclusions

This study aims to evaluate the environmental impacts of producing HIPS and ABS from WEEE plastics through a LCA. To address the research question, scenarios were developed for sensitivity analysis. These scenarios included reductions in energy consumption, increases in transportation distance, shifts to renewable energy sources, and different allocation criteria (mass-based and economic). The results showed that extrusion is the dominant contributor to environmental impacts, while transport plays a minor role. Improvements in energy efficiency and the use of Brazil’s renewable-rich electricity mix significantly reduced impacts, reinforcing that energy-related measures are critical to maximizing environmental benefits. Moreover, recycling consistently demonstrated lower burdens compared to virgin plastic production, highlighting its relevance as a strategy for climate change mitigation and sustainable materials management in Brazil.

Analyzing the potential environmental impacts of obtaining HIPS and ABS from the recycling of WEEE reveals that the primary critical point is extrusion, due to its intensive energy use compared to other processes. Therefore, it is essential to develop strategies that make extrusion less impactful, as reducing energy consumption significantly decreases potential environmental impacts. Optimizing elements of the process, such as heat exchangers, can be a promising approach. Additionally, long-term planning for a structural shift in the energy mix toward renewable sources is important to reduce the reliance on fossil fuels.

In contrast, collection and transportation represent a less significant concern compared to the energy consumption within the recycling plant. It contributes a small portion of the total impact, and large increases in the distance traveled result in proportionally smaller increases in environmental impacts.

Thus, by implementing solutions based on these results, it is possible to reduce the environmental impacts of the WEEE plastics recycling process, promoting a more environmentally sustainable process. This also enables the reuse of materials already present in the technosphere, avoiding the extraction of virgin materials for primary production. These findings also contribute to bridging the gap between process-level LCA studies and policy-oriented circular economy strategies, particularly in emerging economies such as Brazil. In addition, this study contributes to advancing process-level understanding of environmental hotspots in WEEE plastic recycling, particularly in emerging economies, where such detailed analyses remain limited.

Future research should investigate the economic viability of these energy optimization strategies and the broader consequences of transitioning to renewable energy sources. Additionally, examining the effects of various allocation methods in LCA can enhance the precision and relevance of environmental impact evaluations.

## Supplementary Information

Below is the link to the electronic supplementary material.

ESM 1 (DOCX 26.2 KB)

## Data Availability

All data generated or analyzed during this study are included in this published article.

## References

[CR1] Business Analytiq (2025) ABS price index. Available at: https://businessanalytiq.com/procurementanalytics/index/abs-price-index/. Accessed: 22 Feb 2026

[CR2] Andrianisa HA, Sossou SK, Zorom M, Nare L, Ahossouhe MS, Sanou A (2024) An alternative classification approach for waste electronic and electrical equipment (WEEE) recovery in low-income countries: case study in Burkina Faso. Environ Sci Pollut Res 31:39318–39330. 10.1007/s11356-024-33796-8 10.1007/s11356-024-33796-8 38814561

[CR3] Araújo MG, Magrini A, Mahler CF, Bilitewski B (2012) A model for estimation of potential generation of waste electrical and electronic equipment in Brazil. Waste Manag 32:335–342. 10.1016/j.wasman.2011.09.020 22014584 10.1016/j.wasman.2011.09.020

[CR4] Ardolino F, Cardamone GF, Arena U (2021) How to enhance the environmental sustainability of WEEE plastics management: an LCA study. Waste Manag 135:347–359. 10.1016/j.wasman.2021.09.021 34600293 10.1016/j.wasman.2021.09.021

[CR5] Baldé CP, Kuehr R, Yamamoto T, McDonald R, Althaf S, Bel G, Deubzer O, Fernandez-Cubillo E, Forti V, Gray V, Herat S, Honda S, Iattoni G, Khetriwal DS, Luda di Cortemiglia V (2024) Global E-waste Monitor 2024. International Telecommunication Union (ITU) e United Nations Institute for Training and Research (UNITAR), Genebra/Bonn

[CR6] Borrirukwisitsak S, Khwamsawat K, Leewattananukul S, Rewlay-ngoen C (2023) Material flow analysis and life cycle assessment of WEEE dismantling into recycled materials in Thailand. J Mater Cycles Waste Manag 25:3674–3689. 10.1007/s10163-023-01789-3

[CR7] Brasil (2010) Lei no 12.305/2010 – Política Nacional de Resíduos Sólidos (PNRS). Congresso Nacional, Brasília

[CR8] Brasil (2020) Decreto no 10.240/2020 – Logística Reversa de Produtos Eletroeletrônicos. Presidência da República, Brasília

[CR9] Brasil (2024a) Estratégia Nacional de Economia Circular

[CR10] Brasil (2024b) Lei de Incentivo à Reciclagem

[CR11] Cesaro A, Gallo M, Moreschi L, Del Borghi A (2024) The hydrometallurgical recovery of critical and valuable elements from WEEE shredding dust: process effectiveness in a life cycle perspective. Resour Conserv Recycl 206. 10.1016/j.resconrec.2024.107609

[CR12] El-Sherif DM, Abouzid M, Saber AN, Hassan GK (2024) A raising alarm on the current global electronic waste situation through bibliometric analysis, life cycle, and techno-economic assessment: a review. Environ Sci Pollut Res Int 31:40778–40794. 10.1007/s11356-024-33839-0 38819510 10.1007/s11356-024-33839-0

[CR13] European Environment Agency (EEA) (2024) Plastics – circularity sectoral module. Available at: https://www.eea.europa.eu/en/circularity/sectoral-modules/plastics. Accessed: 22 Feb 2026

[CR14] European Union (2003) Directive 2002/95/EC of the European Parliament and of the Council of 27 Jan 2003 on the restriction of the use of certain hazardous substances in electrical and electronic equipment. European Parliament & Council, Bruxelas

[CR15] European Union (2003) Directive 2002/96/EC of the European Parliament and of the Council on Waste Electrical and Electronic Equipment. European Parliament & Council, Bruxelas

[CR16] European Union Joint Research Centre (EU-JRC) (2018) Product Environmental Footprint Category Rules Guidance (PEF) – Version 6.3. 1–238

[CR17] Finnveden G, Hauschild MZ, Ekvall T, Guinée J, Heijungs R, Hellweg S, Koehler A, Pennington D, Suh S (2009) Recent developments in life cycle assessment. J Environ Manage 91:1–21. 10.1016/j.jenvman.2009.06.018 19716647 10.1016/j.jenvman.2009.06.018

[CR18] Gaikwad V, Ghose A, Cholake S, Rawal A, Iwato M, Sahajwalla V (2018) Transformation of E-waste plastics into sustainable filaments for 3D printing. ACS Sustain Chem Eng 6:14432–14440. 10.1021/acssuschemeng.8b03105

[CR19] Garcia FL, Nunes AO, Martins MG, Belli MC, Saavedra YMB, Silva DAL, Moris V (2021) Comparative LCA of conventional manufacturing vs. additive manufacturing: the case of injection moulding for recycled polymers. Int J Sustain Eng 14:1604–1622. 10.1080/19397038.2021.1990435

[CR20] Ghiga SC, Simion IM, Filote C, Roșca M, Hlihor RM, Gavrilescu M (2023) Comparative analysis of three WEEE management scenarios based on LCA methodology: case study in the municipality of Iasi, Romania. Processes. 10.3390/pr11051305

[CR21] Hirayama D, Saron C (2018) Morphologic and mechanical properties of blends from recycled acrylonitrile-butadiene-styrene and high-impact polystyrene. Polymer 135:271–278. 10.1016/j.polymer.2017.12.038

[CR22] International Organization for Standardization (2006a) ISO 14040: Environmental management – Life cycle assessment – Principles and framework

[CR23] International Organization for Standardization (2006b) ISO 14044: Environmental management – Life cycle assessment – Requirements and guidelines

[CR24] Islam MT, Iyer-Raniga U (2023) Life cycle assessment of e-waste management system in Australia: case of waste printed circuit board (PCB). J Clean Prod. 10.1016/j.jclepro.2023.138082

[CR25] Ismail H, Hanafiah MM (2021) Evaluation of e-waste management systems in Malaysia using life cycle assessment and material flow analysis. J Clean Prod. 10.1016/j.jclepro.2021.127358

[CR26] Liu K, Tan Q, Yu J, Wang M (2023) A global perspective on e-waste recycling. Circ Econ 2. 10.1016/j.cec.2023.100028

[CR27] Mendes Campolina J, São Leandro Sigrist C, Faulstich de Paiva JM, Oliveira Nunes A, da Silva Moris VA (2017) A study on the environmental aspects of WEEE plastic recycling in a Brazilian company. Int J Life Cycle Assess 22:1957–1968. 10.1007/s11367-017-1282-2

[CR28] Metaloop (2025) Scrap metal prices. Available at: https://www.metaloop.com/scrap-metal-price/. Accessed: 22 Feb 2026

[CR29] Paz Metals (2025) Scrap prices. Available at: https://pazmetals.com/scrap-prices/#material-5. Accessed: 22 Feb 2026

[CR30] Mulya KS, Zhou J, Phuang ZX, Laner D, Woon KS (2022) A systematic review of life cycle assessment of solid waste management: methodological trends and prospects. Sci Total Environ. 10.1016/j.scitotenv.2022.154903 10.1016/j.scitotenv.2022.154903 35367543

[CR31] Nanda S, Berruti F (2021) Thermochemical conversion of plastic waste to fuels: a review. Environ Chem Lett 19:123–148. 10.1007/s10311-020-01094-7

[CR32] Nikolic M, Bergmann G, Schelte N, Severengiz S (2024) Closing the loop of small WEEE – life cycle based approach for the evaluation of end-of-life strategies on the example of coffee machines. Resour Conserv Recycl Adv 23. 10.1016/j.rcradv.2024.200220

[CR33] Palanisamy K, Subburaj RG (2023) Integration of electronic waste management: a review of current global generation, health impact, and technologies for value recovery and its pertinent management technique. Environ Sci Pollut Res 30:63347–63367. 10.1007/s11356-023-26719-6 10.1007/s11356-023-26719-6 37058236

[CR34] Pokhrel P, Lin SL, Tsai CT (2020) Environmental and economic performance analysis of recycling waste printed circuit boards using life cycle assessment. J Environ Manage. 10.1016/j.jenvman.2020.111276 10.1016/j.jenvman.2020.111276 32871467

[CR35] Pryshlakivsky J, Searcy C (2021) Life cycle assessment as a decision-making tool: practitioner and managerial considerations. J Clean Prod. 10.1016/j.jclepro.2021.127344

[CR36] Rabbi MF, Popp J, Máté D, Kovács S (2022) Energy security and energy transition to achieve carbon neutrality. Energies. 10.3390/en15218126

[CR37] Rocha TB, Penteado CSG (2021) Life cycle assessment of a small WEEE reverse logistics system: case study in the Campinas Area, Brazil. J Clean Prod. 10.1016/j.jclepro.2021.128092

[CR38] Rogers D, Tibben-Lembke R (1998) Going Backwards: Reverse Logistics Trends and Practices. University of Nevada, Reverse Logistics Executive Council, Reno, NV

[CR39] Spirio A, Arrigo R, Frache A, Tuccinardi L, Tuffi R (2024) Plastic waste recycling in additive manufacturing: recovery of polypropylene from WEEE for the production of 3D printing filaments. J Environ Chem Eng. 10.1016/j.jece.2024.112474

[CR40] Sun L, Dong H, Dai Y, Dong J, Fujii M, Geng Y, Lou Z, Liu X (2024) Environmental benefit of recycling plastics from waste electrical & electronic equipment. Resour Conserv Recycl 211. 10.1016/j.resconrec.2024.107855

[CR41] Teixeira FdaSM, Peres ACdeC, Pacheco EBAV (2023) Mechanical recycling of acrylonitrile-butadiene-styrene copolymer and high impact polystyrene from waste electrical and electronic equipment to comply with the circular economy. Front Sustain. 10.3389/frsus.2023.1203457

[CR42] Tutton CG, Young SB, Habib K (2022) Pre-processing of e-waste in Canada: case of a facility responding to changing material composition. Resour Environ Sustain 9. 10.1016/j.resenv.2022.100069

[CR43] Twagirayezu G, Irumva O, Huang K, Xia H, Uwimana A, Nizeyimana JC, Manzi HP, Nambajemariya F, Itangishaka AC (2022) Environmental effects of electrical and electronic waste on water and soil: a review. Pol J Environ Stud 31:2507–2525. 10.15244/pjoes/144194

[CR44] Weidema BP, Bauer C, Hischier R, Mutel C, Nemecek T, Reinhard J, Vadenbo CO, Wernet G (2013) Overview and methodology: Data quality guideline for the ecoinvent database version 3 (Ecoinvent Report No. 1(v3)). The ecoinvent Centre, St. Gallen

[CR45] Xia J, Huang Y, Li Q, Xiong Y, Min S (2021) Convolutional neural network with near-infrared spectroscopy for plastic discrimination. Environ Chem Lett 19:3547–3555. 10.1007/s10311-021-01240-9

