# Supplementary material for: Towards environmental management of WEEE in Brazil: evaluating the impacts of recycling plastics
Source: Environ Sci Pollut Res Int. 2026 Apr 16;33(15):6918–36. doi: 10.1007/s11356-026-37732-w (PMC13156206; doi:10.1007/s11356-026-37732-w)
Supplement: Supplementary file 1 — (DOCX 26.2 KB) [file 11356_2026_37732_MOESM1_ESM.docx]

**Table** **S1:** Economic factors calculation

| **WEEE fraction** | **Mass share (%)** | **Dismantling value (EUR/t)** | **Commodity market value (USD/t)** | **Allocation factor (dismantling – scrap-based)** | **Allocation factor (commodity-based)** |
| --- | --- | --- | --- | --- | --- |
| Iron and steel | 41 | 160 | 434.06 | 0.0894 | 0.0553 |
| Plastics (other than ABS/HIPS) | 17 | 467 | 1,890 | 0.1100 | 0.1000 |
| Plastics – ABS and HIPS | 20 | 467 | 1,890 | 0.1273 | 0.1174 |
| Aluminum | 7 | 1,758 | 2,888.85 | 0.1677 | 0.0628 |
| Components and residues for co-processing | 5 | 0 | 0 | 0 | 0 |
| Copper | 3 | 8,310 | 11,807 | 0.3397 | 0.1100 |
| Residues sent to landfill | 3 | 0 | 0 | 0 | 0 |
| Printed circuit boards (PCBs) | 3 | 4,105.15 | 59,233.30 | 0.1678 | 0.5517 |
| Glass | 1 | 4.31 | 1,040 | 0.00006 | 0.0032 |

**Table S2**: Printed Circuit Board composition

| **PCB component** | **Mass share (%)** | **Market value (USD/t)** |
| --- | --- | --- |
| Copper | 23.67 | 11,807 |
| Lead | 1.60 | 1,999 |
| Nickel | 3.34 | 149,000 |
| Silver | 0.56 | 1,860,000 |
| Gold | 0.02 | 134,570,000 |
| Palladium | 0.03 | 47,000,000 |
| Epoxy resin | 30.70 | – |
| Silica | 31.00 | – |
| Other materials | 9.00 | – |

**Table S3**: Pedigree matrix scores and justification for uncertainty analysis

| **Flow** | **Reliability** | **Completeness** | **Temporal correlation** | **Geographical correlation** | **Technological correlation** | **Justification** |
| --- | --- | --- | --- | --- | --- | --- |
| Diesel for WEEE transportation | 1 | 2 | 1 | 1 | 1 | Primary data obtained directly from company records on fuel consumption, ensuring high reliability and representativeness of the studied system. |
| Carbon dioxide from transportation | 2 | 2 | 1 | 1 | 2 | Emissions estimated based on fuel consumption using established emission factors (e.g., GHG Protocol), introducing moderate uncertainty. |
| dinitrogen monoxide from transportation | 2 | 2 | 1 | 1 | 2 | Estimated using emission factors from standard methodologies, resulting in moderate uncertainty compared to direct measurements. |
| Methane from transportation | 2 | 2 | 1 | 1 | 2 | Calculated using emission factors from established guidelines, leading to moderate reliability. |
| Carbon dioxide, non-fossil from transportation | 2 | 2 | 1 | 1 | 2 | Estimated based on standard emission factors, introducing additional uncertainty compared to directly measured data. |
| WEEE arriving at the company | 1 | 2 | 1 | 1 | 1 | Measured directly from company operational records, ensuring high reliability and representativeness. |
| WEEE sent to the disassembly and separation area | 1 | 2 | 1 | 1 | 1 | Based on internal process data from the company, with direct measurement and high consistency. |
| WEEE plastics in disassembly and separation area | 1 | 2 | 1 | 1 | 1 | Derived from company data on material composition and separation processes, ensuring high data quality. |
| Other WEEE components | 1 | 2 | 1 | 1 | 1 | Obtained from company records and internal mass balance, ensuring high reliability. |
| WEEE-derived ABS/HIPS plastics to be ground | 1 | 2 | 1 | 1 | 1 | Measured directly within the company's process flow, ensuring high technological and geographical representativeness. |
| Ground WEEE-derived ABS/HIPS plastics to be extruded | 1 | 2 | 1 | 1 | 1 | Based on direct process measurements and internal tracking, ensuring high reliability. |
| ABS/HIPS pellets | 1 | 2 | 1 | 1 | 1 | Final product output measured directly in the production process, ensuring high data quality. |
| Extrusion waste | 1 | 2 | 1 | 1 | 1 | Calculated based on process yields and internal measurements, ensuring high representativeness. |
| Waste treatment | 2 | 2 | 1 | 1 | 1 | Based on secondary data from LCA databases (e.g., Ecoinvent), introducing moderate uncertainty despite alignment with standard practices. |
| Energy – disassembly and separation | 2 | 2 | 1 | 1 | 1 | Estimated based on equipment power ratings and operational time, introducing moderate uncertainty compared to direct measurement. |
| Energy - grinding | 2 | 2 | 1 | 1 | 1 | Calculated using equipment specifications and operational time, resulting in moderate reliability. |
| Energy - extrusion | 2 | 2 | 1 | 1 | 1 | Estimated from machine power and operating conditions, introducing some uncertainty due to process variability. |
| Energy - stock | 2 | 2 | 1 | 1 | 1 | Based on estimated consumption from facility operations, with moderate uncertainty. |
